# Diagnosis and management of rare acute erythroid leukemia with hemophagocytic lymphohistiocytosis: a case report

**DOI:** 10.3389/fonc.2026.1768823

**Published:** 2026-02-16

**Authors:** Yan Zhang, Gusheng Tang, Hui Cheng

**Affiliations:** Department of Hematology, Changhai Hospital, Naval Medical University, Shanghai, China

**Keywords:** acute erythroid leukemia, diagnosis, hemophagocytic lymphohistiocytosis, prognosis, therapy

## Abstract

**Introduction:**

Acute erythroid leukemia (AEL) complicated by hemophagocytic lymphohistiocytosis (HLH) is an exceedingly rare hematologic malignancy. Its diagnosis relies on a comprehensive assessment that includes bone marrow cytomorphology, immunophenotyping, cytogenetics, molecular profiling, and serum ferritin levels. Its management poses substantial clinical challenges, and the prognosis is generally guarded.

**Main symptoms and/or important clinical findings:**

The patient was admitted due to persistent fatigue for 20 days and recurrent fever. A complete blood count showed pancytopenia: white blood cells (2.99 ×10^9^/L), red blood cells (2×10¹²/L), hemoglobin (70g/L), and platelets (15×10^9^/L). Ferritin levels exceeded 2000µg/L, lactate dehydrogenase (LDH) was elevated to 1414 U/L and triglyceride was normal. The coagulation profile indicated normal fibrinogen levels; however, its degradation product was elevated (7.70μg/mL), along with increased plasma D-dimer (1.48μg/mL). Elevated inflammatory markers included C-reactive protein (33.90mg/L) and procalcitonin (1.400ng/mL). A non-contrast computed tomography (CT) scan revealed bilateral pulmonary inflammatory exudation, atelectasis, and splenomegaly.

**The main diagnoses, therapeutic interventions, and outcomes:**

Comprehensive bone marrow evaluation confirmed a diagnosis of AEL complicated by secondary HLH. Initial therapy with a decitabine-CAG (aclacinomycin, cytarabine, G-CSF)-venetoclax regimen failed to induce remission. Morphological complete remission was achieved after switching to a DAE (daunorubicin, cytarabine, etoposide) regimen. Despite plans for allogeneic hematopoietic stem cell transplantation, the patient succumbed within 3 months of diagnosis.

**Conclusion:**

This case highlights the diagnostic and therapeutic complexities associated with the co-occurrence of AEL and HLH. Early identification of HLH as a potential complication in AEL is crucial, though outcomes remain dismal, emphasizing an urgent need for novel therapeutic strategies.

## Introduction

Acute erythroid leukemia (AEL) is a subtype of acute myeloid leukemia (AML). According to the 2022 WHO classification criteria, the diagnosis requires that erythroid precursors constitute >80% of all bone marrow nucleated cells, with at least 30% being proerythroblasts (cases with erythroid precursor < 80% may also be recognized under specific diagnostic circumstances) (1). AEL is frequently characterized by complex karyotypes and *TP53* mutations (2, 3).

Hemophagocytic lymphohistiocytosis (HLH) is a highly aggressive syndrome characterized by a high risk of multi-organ failure and mortality if not promptly treated. It is broadly classified into primary (genetic) and secondary (acquired) forms (4). The incidence of primary HLH demonstrates considerable geographical variation. In comparison, studies indicate that severe infections are a significant trigger for secondary HLH, and approximately 8% of cases develop as complications of leukemia (5, 6). Nevertheless, HLH occurring secondary to AEL is a relatively rare phenomenon.

The current first-line treatment strategy for AEL mainly involves intensive chemotherapy (ICT) and hypomethylating agents (HMAs). Although allogeneic bone marrow transplantation is a potentially curative approach, it requires achieving complete remission prior to initiation (7). The management of HLH follows the principle of timely control of hyperinflammation and actively identifying potential triggers. The current treatment remains the etoposide-based HLH-94 and HLH-2004 regimens as standard methods (8, 9). Here, we report a rare case of AEL complicated by HLH, highlighting significant therapeutic challenges. Early diagnosis and prompt individualized treatment may improve the outcomes of such patients.

## Case presentation

A 63-year-old man presented with persistent fatigue for 20 days and recurrent fever. No systemic lymphadenopathy was detected. The patient denied any history of autoimmune diseases, neurological disorders, or other pre-existing medical conditions. The serum ferritin level was markedly elevated (>2000µg/L), lactate dehydrogenase (LDH) was increased to 1414 U/L and triglyceride was normal. Coagulation profile showed a normal fibrinogen level, but elevated fibrinogen degradation products (7.70µg/mL) and plasma D-dimer (1.48µg/mL). Inflammatory markers were also elevated, including C-reactive protein (33.90mg/L) and procalcitonin (1.400ng/mL). CT scan demonstrated bilateral pulmonary inflammatory infiltrates, atelectasis, and splenomegaly.

**Figure 1:**
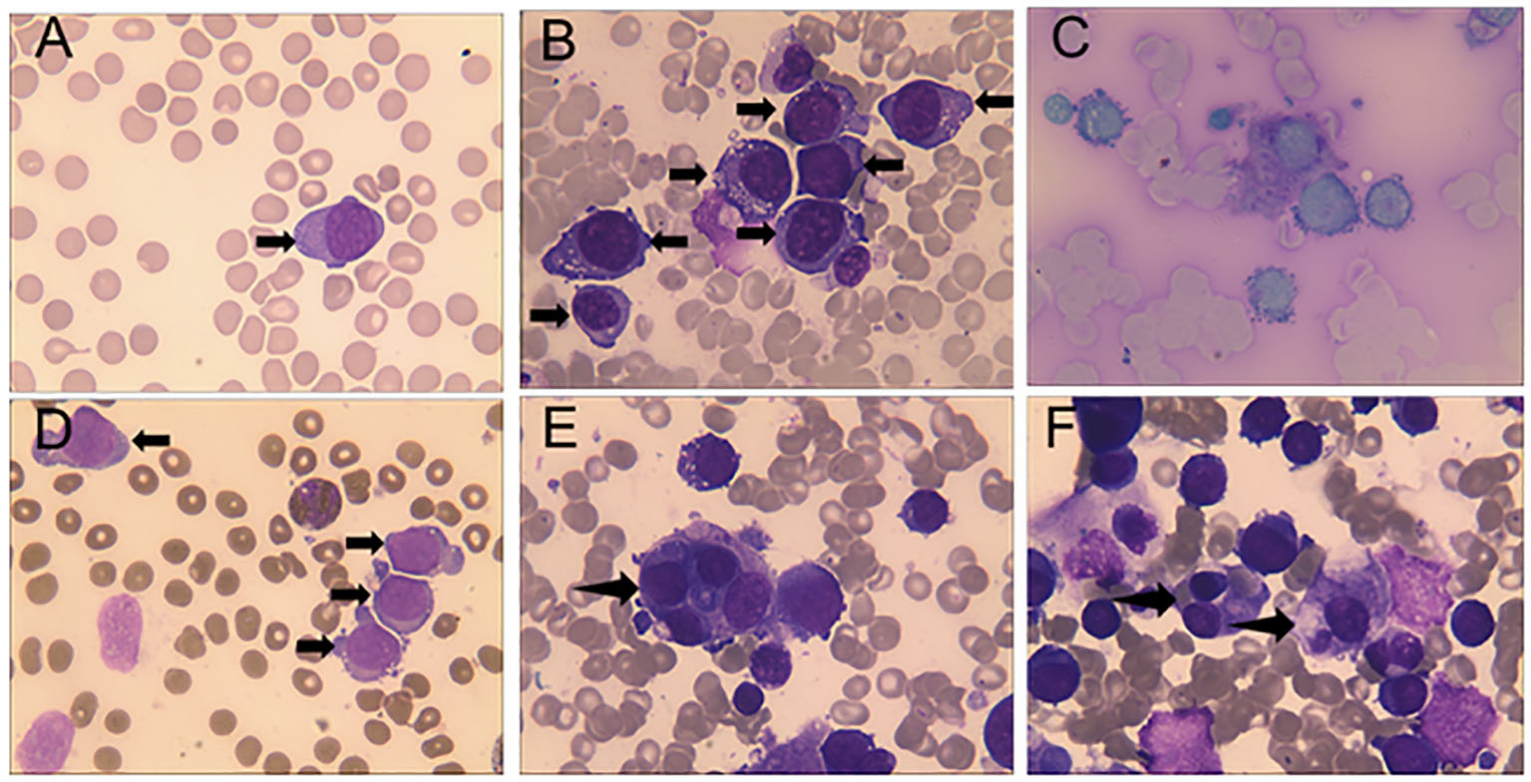
Peripheral blood shown proerythroblasts and bone marrow aspirate revealed numerous proerythroblasts and hemophagocytic histiocytes. **(A)** Peripheral blood smear (Wright-Giemsa stain, ×1000) showing proerythroblasts. **(B)** Bone marrow smear (Wright-Giemsa stain, ×1000) showing numerous proerythroblasts and hemophagocytic histiocytes. **(C)** Bone marrow smear (Periodic acid-Schiff stain, ×1000), coarse granules were visible within the cytoplasm, predominantly distributed perinuclearly in proerythroblasts. **(D)** Bone marrow smear (myeloperoxidase stain, ×1000), proerythroblasts were peroxidase-negative, and the control granulocytes shown a granular positivity in the cytoplasm. **(E, F)** Bone marrow smear (Wright-Giemsa stain, ×1000), hemophagocytic histiocytes engulfing erythroid precursors were observed.

**Figure 2:**
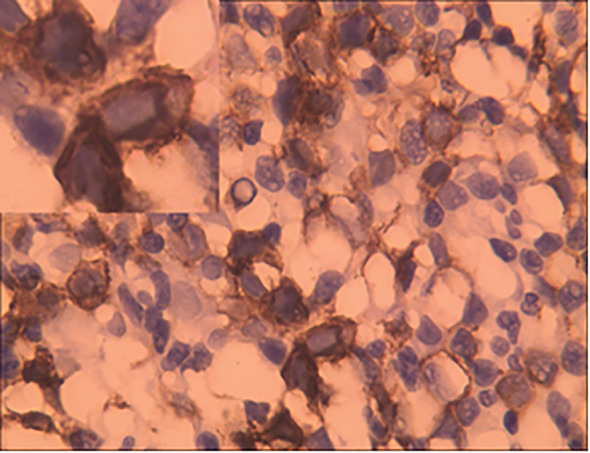
Bone marrow trephine biopsy (immunohistochemistry, ×400) shown leukemic cells with expression of the erythroid marker E-cadherin.

The peripheral blood smear of this patient demonstrates 3% proerythroblasts, while the bone marrow smears (BMS) revealed hypercellularity, 70.5% of the cells being erythrocytes, with 53% proerythroblasts having large round nuclei, dispersed chromatin, 1 to 3 nucleoli, deeply basophilic cytoplasm with vacuoles, and high nuclear-to-cytoplasmic ratios (**Figures 1A,B**). These blasts demonstrated positive staining for the periodic acid-Schiff (PAS) reaction (**Figure 1B**), while the myeloperoxidase (MPO) staining was negative (**Figure 1C**). Simultaneously, numerous hemophagocytic histiocytes were observed on BMS (**Figures 1D,E**). Flow cytometry identified a monoclonal erythrocyte population, and cytogenetic demonstrated a complex karyotype with 43-44, X, -Y, del (5) (q13q31), del (7) (q22q34), -13, add (14) (pter), -19, -20, add (22) (qter), +mar, inc[cp15]. Molecular analysis detected a p. Cys238Tyr (c. 713G>A) somatic mutation of *TP53* with a variant allele frequency of 33.7% and *WT1* mRNA overexpression (59%, cutoff value <0.56%). Bone marrow biopsy demonstrated sheets of neoplastic erythroblasts (>80% cellularity), immunohistochemically E-cadherin positive (**Figure 2**), which is crucial for confirming erythroid lineage (10).

Treated with a combination of decitabine(25mg, d1-5) plus CAG regimen (aclacinomycin 10mg/m^2^ d3-6, cytarabine 20mg/m^2^/12h d3-10, G-CSF 300u/m^2^/d) with venetoclax (200mg/m^2^/d) failed to achieve remission, after which it was changed to the DAE regimen (daunorubicin 80mg/m^2^ d1-3, cytarabine 170mg/m^2^ d1-7, etoposide 100mg/m^2^ d1-7). After chemotherapy, the patient experienced a period of bone marrow hematopoietic suppression, marked by significant thrombocytopenia and recurrent fever. Blood culture revealed the presence of Gram-positive cocci (staphylococcus epidermidis). Consequently, the patient was administered platelet transfusions to prevent hemorrhage, along with glucocorticoids and antibiotics (meropenem, tigecycline and vancomycin) to control body temperature and provide anti-inflammatory therapy. Subsequent sternal bone marrow aspiration confirmed remission. However, following chemotherapy, the patient developed a pulmonary infection with recurrent high fever. The platelet count was critically low, accompanied by hemorrhages in the conjunctiva and sclera of both eyes, oral mucosal bleeding, a pharyngeal hematoma, widespread petechiae and ecchymoses over the body, and hematuria, manifesting a severe bleeding tendency. The patient ultimately died from hemorrhagic shock and respiratory failure, with the entire disease course lasting only three months.

## Discussion

Acute erythroid leukemia (AEL) is a type of leukemia characterized by generally non-specific clinical manifestations. The primary symptoms typically include fever, pallor, anemia, hepatosplenomegaly, and hemolysis (11). The diagnosis and prognostic assessment of AEL primarily rely on bone marrow cell morphology, immunophenotyping, cytogenetics, and molecular biology, often in combination with bone marrow biopsy. Bone marrow examination findings (cytomorphology, histopathology, and erythroid antigen immunohistochemistry) met the diagnostic criteria for AEL.

The potential pathogenesis of AEL encompasses erythropoietin receptor (*EPOR*) activation (12), low expression of *GATA1 (*13), overexpression of *c-myc* (14), *TP53* mutation (15) and epigenetic dysregulation (16), among others. Although some underlying mechanisms of AEL have been identified, effective treatment options remain limited. The current first-line treatment for AEL involves intensive chemotherapy and hypomethylating agents. Allogeneic hematopoietic stem cell transplantation represents the only potentially curative approach (7). The prognosis of AEL is dismal, with a median survival of 3–9 months (17). In this case, the patient failed to achieve remission with intensive CAG-venetoclax chemotherapy and was subsequently switched to the DAE regimen. Although post-treatment bone marrow morphology indicated remission, the patient survived only 3 months, which may be attributed to a combination of treatment-related complications, the complex karyotype and *TP53* mutation of AEL, and secondary HLH.

Secondary HLH is commonly seen in adults, primarily caused by severe inflammation, tumors, or autoimmune diseases (5). The diagnosis of HLH is primarily based on the HLH-2004 criteria (fulfilling 5/8 criteria: splenomegaly, fever, cytopenias, hypertriglyceridemia and/or hypofibrinogenemia, hemophagocytosis, low or absent NK-cell activity, hyperferritinemia and high levels of sIL-2r). The main symptoms and clinical findings of this patient met 5 of these criteria. Notably, NK cell activity and sIL-2R levels were not tested and triglyceride and/or fibrinogen levels were within the normal range. If a molecular diagnosis consistent with HLH is established, meeting the full set of these clinical criteria may not be obligatory (18). The pathogenesis of secondary HLH is also multifactorial, involving converging triggers that cause uncontrolled inflammation (19). However, its exact mechanisms are not fully understood.

For the management of secondary HLH, therapeutic options beyond etoposide and glucocorticoids include interleukin-1 inhibitors (anakinra), interferon-gamma inhibitors (emapalumab), Janus kinase inhibitors (ruxolitinib), and intravenous immunoglobulin (4). Despite the administration of glucocorticoids and etoposide, the therapeutic response in this patient was suboptimal. This may be related to poor tolerance to the combined therapeutic burden, given the concurrent intensive chemotherapy for AEL. There have been reports indicating that the ruxolitinib combined with dexamethasone yielded a rapid therapeutic response with a favorable side-effect profile for treatment of secondary HLH (20). Regrettably, this regimen could not be administered to our patient in time.

## Conclusion

AEL accounts for less than 1% of AML, with cases complicating HLH being distinctly rare (1, 21). Herein we report a case of AEL characterized by a complex karyotype and *TP53* mutation. At diagnosis, a significant number of hemophagocytes were observed on the bone marrow smear. Based on this finding and the patient’s clinical manifestations, a diagnosis of AEL with secondary HLH was established. The morphological and genetic features of AEL indicate that it is a subtype of AML with a dismal prognosis, necessitating the formulation of corresponding individualized treatment strategies (2). Understanding the etiology of HLH can effectively assist clinicians in making an early diagnosis and implementing timely interventions (22). This case underscores the catastrophic outcome of rare AEL-HLH convergence, which indicates significant therapeutic challenges from managing both a highly refractory malignancy and HLH.

## Data Availability

The raw data supporting the conclusions of this article will be made available by the authors, without undue reservation.
